# Effects of chronic widespread pain on the health status and quality of life of women after breast cancer surgery

**DOI:** 10.1186/1477-7525-3-30

**Published:** 2005-04-28

**Authors:** Carol S Burckhardt, Kim D Jones

**Affiliations:** 1 Primary Care, School of Nursing, Oregon Health & Science University, Portland, Oregon, USA

## Abstract

**Background:**

Most research and treatment of post-breast cancer chronic pain has focused on local or regional pain problems in the operated area. The purpose of this pilot study was to compare and contrast the pain characteristics, symptom impact, health status, and quality of life of post-breast cancer surgery women with regional chronic pain versus those with widespread chronic pain.

**Methods:**

A cross-sectional, descriptive design compared two groups of women with chronic pain that began after surgery: regional pain (n = 11) and widespread pain (n = 12). Demographics, characteristics of the surgery, as well as standardized questionnaires that measured pain (Brief Pain Inventory (BPI), Short Form McGill Pain Questionnaire (MPQ-SF)), disease impact (Fibromyalgia Impact Questionnaire (FIQ), Functional Assessment of Cancer Therapy-Breast (FACT-B)), health status (Medical Outcomes Short Form (SF-36)) and quality of life (Quality of Life Scale (QOLS)) were gathered.

**Results:**

There were no significant differences between the groups on any demographic or type of surgery variable. A majority of both groups described their pain as aching, tender, and sharp on the MPQ-SF. On the BPI, intensity of pain and pain interference were significantly higher in the widespread pain group. Differences between the two groups reached statistical significance on the FIQ total score as well as the FACT-B physical well-being, emotional well-being and breast concerns subscales. The SF-36 physical function, physical role, and body pain subscales were significantly lower in the widespread pain group. QOLS scores were lower in the widespread pain group, but did not reach statistical significance.

**Conclusion:**

This preliminary work suggests that the women in this study who experienced widespread pain after breast cancer surgery had significantly more severity of pain, pain impact and lower physical health status than those with regional pain.

## Background

Breast cancer is the most common form of cancer among women in the United States, Canada and Europe [1,2]. A sharp increase in incidence has been seen over the past two decades due in large part to use of mammography and subsequent earlier detection of disease. Earlier detection and treatment has led to increased survival rates approaching 90% for noninvasive cancers [3,4] Thus, a large majority of women with breast cancer will survive for many years after the initial diagnosis and treatment. Because they are living longer, living well with a good quality of life has become a high priority [5,6].

A recent study of health-related quality of life (HRQOL) concluded that most women treated for early-stage breast cancer have generally high HRQOL when compared to norms for the general population [7]. Unfortunately, long-term disease and treatment-related symptoms, such as chronic pain, can have wide-ranging consequences for health, functioning, and life quality [8-10]. Several researchers have reported that a substantial number of long-term breast cancer survivors experience chronic pain that interferes with physical functioning, mood, work, relationships, sleep, and enjoyment of life [11-15].

Chronic localized or regional pain after breast cancer surgery is a common and well-recognized problem with prevalence rates ranging from 20 to 65% [16-18]. Much of this pain is believed to be neuropathic phenomena due to transsection of nerves during surgery, nerve entrapment, axillary hematoma, or development of a traumatic neuroma on the operated side [19-21]. In addition, significantly higher rates of chronic pain on the affected side have been reported in patients who have had a mastectomy with reconstruction versus those who have had a mastectomy alone [18] and those who have undergone extensive axillary node dissection [22,23].

However, growing evidence exists that neuropathic phenomena alone do not explain all of the chronic pain experienced by post-surgery patients. Many breast cancer patients suffer from widespread, diffuse persistent pain that may be due to the chronic activation of nociceptors [24,25]. Reports on mastectomy with or without breast reconstruction have found a significant risk for development of fibromyalgia (FMS) [26], a specific syndrome of widespread chronic pain that affects approximately 2–5% of the female adult populations in the United States and many other countries [27,28].

Recent evidence has documented central sensitivity due to chronic input from peripheral nerves as a major cause of chronic pain in FMS patients [29,30]. And further, that trauma, especially to the upper body, is more likely to eventually lead to diffuse, widespread pain [31]. In both breast cancer patients and FMS patients, upper body trauma may be a factor in the onset and persistence of chronic widespread pain [32,33]. These findings are important because dysfunction, disability and detriments to quality of life are substantial in the FMS population [34,35]. But as yet, diffuse, widespread chronic pain among post-surgery breast cancer patients has not received the same attention as localized neuropathic pain.

In summary, until now, most work in post-breast cancer chronic pain has focused on local or regional pain problems in the operated area. Yet, a substantial number of post-breast cancer surgery women may have chronic widely diffuse pain that limits their functioning and decreases their overall life quality. The purpose of this pilot study was to compare and contrast the pain characteristics, syndrome impact, health status, and quality of life of post-breast cancer surgery women with localized or regional chronic pain versus post-breast cancer surgery women with widespread chronic pain.

## Methods

### Design

This pilot project used a cross-sectional, descriptive design. Participants responded to a battery of self-report questionnaires. The project focused on gathering preliminary descriptive data using well-validated scales.

### Participants

Participants were recruited through an advertisement placed in a local newspaper, and mailings containing the advertisement to a database of women with FMS at a university out patient FMS clinic and to a university breast cancer clinic. The target sample size was 30.

The advertisement invited women, who believed that they met the criteria listed below, to call and leave a message at a specified number. An investigator called the woman, answered any questions about the study and then invited the woman to come to the clinic at a specified time if it appeared that she met criteria. Written informed consent for the data collection was obtained at the clinic visit after eligibility requirements were confirmed by the investigator.

### Selection of participants

Women were eligible if they had had a simple mastectomy, lumpectomy, or modified radical mastectomy for breast cancer and included those with expansion or breast reconstruction at the time of initial surgery or subsequent breast reconstruction. Other specific inclusion criteria included: (1) adults at least 18 years of age; (2) first time primary diagnosis of breast cancer; (3) post-mastectomy for at least 6 months; (4) at least 3 months post-primary adjuvant treatment (radiation, cytotoxic chemotherapy); (5) cancer-free by their own report and having seen their physician within the last year; and, (6) current chronic pain by their own report that began after the breast cancer surgery. Exclusion criteria included: (1) breast surgery for cosmetic reasons or prophylactic mastectomy; and, (2) other painful or disabling medical conditions, such as arthritis.

### Data collection

Demographic and cancer history information was obtained using a checklist and short answer questionnaire. Demographic variables included age, ethnic background, marital status, education, employment status, and occupation. Cancer history variables included date of surgery, type of breast cancer surgery, type of breast reconstruction surgery, adjuvant therapy, present cancer status.

### Instruments for measuring pain characteristics

1. **Short Answer Questions **related to location, onset, extent, and quality of the pain as well as any medications for pain.

2. **Body Diagram **developed for use in FMS patients (Chris Henriksson, unpublished data). It was used in this study to distinguish regional from widespread pain. The subject was asked to mark each of 36 segments with a number between 1 and 10 signifying intensity of pain if she had any pain in that segment. This enabled us to distinguish regional pain (limited to 1 or 2 quadrants – upper, lower, right or left) and widespread pain that involved 3 or 4 quadrants as well as obtain some measure of the intensity of the pain at the time of the data collection. The use of quadrants to distinguish regional from widespread pain follows the American College of Rheumatology criteria for the classification of FMS [36].

3. **The Brief Pain Inventory (BPI) **which provides information on intensity (sensory dimension) of pain and the degree to which pain interferes with function (reactive dimension). The BPI uses 0 to 10 numeric rating scales and asks patients to rate their pain at the time of responding to the questionnaire as well as at its worst, least, and average over the previous week. Using the same rating scales, it asks the patient to rate the degree of interference with general activity, mood, walking and other physical activity, work, social activity, relationships with others and sleep. Developed originally for use in cancer treatment and research, the BPI has been well validated in cancer patients [37,38].

4. **The Short-form McGill Pain Questionnaire **a 15-item form of the longer McGill Pain Questionnaire [39]. Each descriptor is rated on a 4-point scale. Evidence for validity in cancer and FMS has been established [40,41]. This questionnaire was used to characterize the similarities and differences in pain descriptions between the two groups.

### Instruments for measuring syndrome impact, health status and quality of life

1. **The Fibromyalgia Impact Questionnaire **a 10-item instrument that measures difficulties with activities of daily living and symptoms of pain, fatigue, morning tiredness, stiffness, job difficulty, depression and anxiety along with amount of work missed and overall well-being during the past week [42]. The instrument has been validated for FMS patients and discriminates between FMS, rheumatoid arthritis and healthy people. It is scored as individual items and as a total score that indicates more FMS impact. This instrument was used to measure overall symptom impact of widespread pain. Scores on the individual items are standardized from 0 to 10 and a total score can range from 0 to 100 with a higher score indicating greater impact (Current scoring information can be obtained at ).

2. **Functional Assessment of Cancer Therapy (FACT-B) **a 44-item self-report questionnaire designed to measure multidimensional health-related quality of life in patients with breast cancer [43]. The FACT-B consists of the FACT-G plus additional items that make up the breast cancer subscale [44]. The FACT-G contains five subscales: physical, functional, social/family and emotional well being and satisfaction with doctors. Each item is rated on a 5-point scale with 0 equal to "not at all" and 4 equal to "very much." Items are reversed if necessary and summed so that a higher subscale score indicates higher well being or satisfaction. All ratings on the FACT-B are completed in terms of the past seven days.

3. **The SF-36 **a 36-item scale that measures 9 domains of health including physical functioning, physical role limitations, bodily pain, general health, vitality, social functioning, emotional role limitations, mental health and change in health [45]. Higher scores indicate better health. The SF-36 has been used in numerous studies of cancer patients and has evidence of validity in both cancer and widespread pain patient groups [46,47]. We used this instrument as the measure of health status.

4. **The Quality of Life Scale (QOLS) **a 16-item non-health focused scale that measures satisfaction with multiple domains of life [48,49]. It uses a 1 to 7 point rating scale anchored with the words terrible and delighted. A higher total score indicates higher quality of life. Use in chronic illness populations, including a small group of cancer patients with ostomies, has been validated.

### Statistical analysis

All data were analyzed using the SPSS version 12 statistical software package. Descriptive statistics (frequencies, means, standard deviations, and percentages) were used to characterize the sample. Preliminary inferences using t-tests for two independent samples and Chi-square for proportions were made. Because normality and equality of variance could not be assumed in the small samples, Q-Q plots and Levene tests were carried out. Q-Q plots revealed no serious deviations from normality. Levene tests for equality of variances enabled us to adjust the significance levels of the t-tests if the assumption of equal variance was not met for a variable. Alpha level for a significant difference was set at 0.01 because of the number of variables. As a check on the validity of the parametric test, we also ran a nonparametric analysis for 2 independent groups (Mann Whitney U) and found the same variables to be significant as on the t-test. Formal directional hypotheses were not made *a priori*. However, we expected to find that the group with widespread pain would have more severe pain impact, poorer health status and lower quality of life than the group with regional pain only.

## Results

In all 30 women replied to the advertisement, 27 expressed initial interest in the study after talking with an investigator by telephone, and 23 scheduled and kept a clinic appointment at the university that reviewed and approved the study, signed the written consent form and completed data collection. No data were collected from the 7 women who inquired about the study but did not sign a consent form. The regional pain group was comprised of 11 women who reported pain only in the upper body (1 or 2 quadrants) while the widespread pain group was comprised of 12 women who reported pain in either 3 or 4 quadrants. The regional pain group marked an average of 2 areas on the body diagram; whereas the widespread pain group marked an average of 12 areas. The demographic, surgery and treatment characteristics of the sample are shown in Table 1. All subjects were at least 1 year post-surgery, radiation and chemotherapy and 80% were within 5 years of initial treatment. Sixty-five percent were on anti-estrogen therapy with either tamoxifen or remedex at the time of the study.

**Table 1:** Demographic and Breast Cancer Surgery and Treatment Characteristics by Pain Extent Group

**Variable**	**Regional Pain **n = 11	**Widespread Pain **n = 12
Age (years)	56.8 (5.5)	58.7 (8.6)
Education (years)	16.0 (2.4)	14.8 (3.2)
Ethnic (% white)	91	100
Marital Status (% married)	73	69
Employment (% employed)	63	67
Time since Surgery (years)	5.9 (2.9)	5.4 (3.6)
Type of Surgery		
Lumpectomy (%)	36	33
Mastectomy (%)	64	67
Axillary Node Dissection (%)	54	67
Breast Reconstruction (%)	18	0
Tissue Expansion (%)	100	0

Radiation Therapy (%)	60	80

Time Since Radiation (years)	4.2 (3.2)	2.9 (3.0)

Chemotherapy (%)	55	64

Time Since Chemo (years)	5.5 (2.4)	2.8 (1.6)

Anti-estrogen Therapy (%)	73	75

Current Anti-estrogen Therapy (%)	36	58

Numbers are means and standard deviations except when noted as percentages

Pain characteristics summarized in Table 2 indicated that a majority in both groups experienced the onset of their chronic pain immediately after surgery with a lesser number noting the onset weeks to months later. Those with regional pain were much more likely to describe their pain as intermittent while those with widespread pain were evenly split in this regard. Those with widespread pain were more likely to rate their pain as worse when depressed or fatigued, and observed a worsening of pain with exercise, rest, cold or contact with clothing. All subjects in the widespread pain group rated medication as at least somewhat effective for relieving their pain. Only 56% of those with regional pain noted any pain relief from medication. Of the 23 subjects, 5 were taking no medications, 12 were on non-steroidal antiinflammatories, 3 were taking narcotic pain relievers, 2 were on neurontin and 1 took glucosamine.

**Table 2:** Pain Characteristics by Pain Extent Group

**Variable**	**Regional Pain **n = 11	**Widespread Pain **n = 12
When did pain begin		
Immediately	63	56
Weeks later	12	11
Months later	25	33
Pain		
Constant	20	50
Intermittent	80	50
Worse when Depressed	22	50
Worse when Fatigued	67	80
Relationship to Activity (Worse)		
Exercise	44	70
Clothing Contact	11	36
Rest	33	73
Cold	25	56
Relief with Medication		
Yes	22	30
Somewhat	34	70
No	44	0

Numbers are percentages

As seen in Table 3, a majority of both groups described their pain as aching, tender, and sharp. A higher percentage of the regional pain group described their pain as throbbing and stabbing while a higher percentage of the widespread pain group described it as shooting, cramping, gnawing, hot-burning, heavy, and splitting. They were also much more likely to use the words that described the emotional components of pain, such as tiring-exhausting and sickening. Overall, those with widespread pain endorsed more items than those with regional pain.

**Table 3:** Short-Form McGill Pain Questionnaire by Pain Extent Group

**Variable**	**Regional Pain **(n = 11)	**Widespread Pain **(n = 12)
Throbbing	27	17
Shooting	46	50
Stabbing	64	42
Sharp	54	58
Cramping	18	33
Gnawing	0	58*
Hot-Burning	9	50
Aching	73	83
Heavy	45	50
Tender	55	67
Splitting	18	33
Tiring-Exhausting	9	67*
Sickening	0	42
Fearful	9	17
Punishing-Cruel	18	33
Total Score	4.4 (2.7)	7.0 (3.7)

Numbers are percent of group that endorsed the item

*<0.01; **<0.001

Intensity of pain as well as multiple measures of pain interference on the BPI were all higher in the widespread pain group with the differences reaching statistical significance on all but three items (Table 4). Results of the FIQ (Table 5) were similar. The widespread pain group had higher impact scores and the differences between the two groups reached statistical significance on 5 of the 10 items as well as the total FIQ score. The FACT-B and SF-36 results shown in Table 6 revealed a statistically significant difference in the two groups on subscales measuring physical health status and well-being. Table 7 lists the items on the QOLS along with the total score. Although most of the scores were lower in the widespread pain group, none reached statistical significance.

**Table 4:** Brief Pain Inventory by Pain Extent Subgroup

**Variable**	**Regional Pain**	**Widespread Pain**
Pain Worst	3.1 (1.9)	6.6 (2.3)**
Pain Least	1.0 (1.3)	3.2 (2.9)
Pain Average	2.7 (1.6)	4.9 (2.1)*
Pain Right Now	1.4 (1.4)	5.2 (2.4)**
Pain Interference		
General Activity	0.6 (1.3)	4.5 (2.1)**
Mood	1.0 (1.8)	4.8 (2.2)**
Walking Ability	0.6 (1.5)	4.2 (2.0)**
Normal Work	1.1 (1.7)	4.9 (2.5)**
Relations with others	0.4 (0.9)	4.2 (2.6)**
Sleep	2.3 (3.2)	5.4 (2.9)
Enjoyment of Life	1.3 (1.0)	5.2 (2.3)**

Numbers are means and standard deviations.

*<0.01; **<0.001

**Table 5:** Fibromyalgia Impact Questionnaire by Pain Extent Subgroup

**Variable**	**Regional Pain**	**Widespread Pain**
Physical Activity	1.5 (2.4)	3.1 (2.4)
Felt Good	1.6 (2.3)	6.1 (3.1)**
Missed Work	0 (0)	1.9 (3.0)
Job Difficulty	1.2 (2.1)	5.2 (2.4)**
Pain	3.5 (2.9)	5.8 (1.5)
Fatigue	4.3 (3.4)	8.0 (1.2)*
Unrested	3.4 (2.6)	7.2 (1.7)**
Stiffness	3.0 (3.3)	5.8 (2.6)
Anxiety	1.1 (2.2)	4.6 (2.8)*
Depression	1.4 (2.4)	4.3 (3.0)
FIQ Total Score	20.9 (13.2)	52.0 (15.1)**

Number are means and standard deviations

*<0.01; **<0.001

**Table 6:** FACT-B and SF-36 by Pain Extent Subgroup

**Variable**	**Regional Pain**	**Widespread Pain**
**FACT-B**		
Physical Well-Being	22.9 (2.9)	16.0 (4.7)**
Social/Family Well-being	19.4 (4.8)	15.7 (6.1)
Relationship with Doctor	6.1 (1.9)	4.8 (2.5)
Emotional Well-being	20.1 (2.8)	15.7 (4.0)**
Functional Well-being	22.1 (3.9)	16.7 (5.7)*
Additional Concerns	24.3 (6.7)	16.5 (5.5)*
**SF-36**		
General Health	60.3 (19.5)	57.9 (21.4)
Physical Functioning	68.6 (23.9)	48.7 (17.1)*
Role-Physical	63.6 (39.3)	18.7 (24.1)*
Bodily Pain	60.4 (14.4)	41.4 (14.1)*
Vitality	41.4 (16.3)	27.1 (14.2)
Social Functioning	78.4 (20.2)	61.4 (22.3)
Role-Emotional	75.7 (30.1)	47.2 (38.8)
Mental Health	61.1 (13.9)	51.3 (14.3)

*<0.01; **<0.001

Numbers are means and standard deviations

**Table 7:** Quality of Life Scale (QOLS) by Pain Extent Subgroup

**Variable**	**Regional Pain**	**Widespread Pain**
Material Comforts	5.0 (1.7)	5.0 (1.6)
Health	4.2 (1.5)	3.2 (1.3)
Relationships with relative	4.8 (1.5)	4.7 (1.2)
Having and rearing children	5.0 (1.3)	5.4 (1.2)
Relationship with spouse or partner	5.5 (1.1)	5.0 (1.9)
Having Close friends	6.0 (0.9)	5.7 (1.4)
Helping Others	5.9 (0.8)	5.3 (1.3)
Civic Activities	5.1 (1.6)	4.8 (1.3)
Intellectual Development	5.7 (1.3)	4.8 (1.1)
Understanding Self	5.4 (0.8)	5.0 (1.3)
Occupational Role	4.9 (1.7)	4.2 (1.4)
Creative expression	5.4 (1.2)	4.2 (1.6)
Socializing	5.4 (1.3)	4.4 (1.6)
Passive recreation	6.2 (0.9)	5.7 (1.4)
Active Recreation	4.7 (1.9)	3.4 (1.1)
Independence	5.9 (1.8)	5.1 (1.1)
QOLS Total Score	85.2 (10.0)	76.0 (15.3)

Numbers are means and standard deviations

## Discussion

The results of this pilot study describe two groups of post breast cancer surgery patients who were similar in demographic, surgery and pain characteristics but markedly different in the impact that pain has on their health status and functioning. Widespread pain, which not been studied as a separate entity in this population, interferes with most aspects of these women's lives to a much greater degree than does regional pain.

For many patients, the chronic pain began immediately after surgery or early in the post-surgical treatment period. This type of onset is well recognized by breast cancer surgeons [23,50] and a number of efforts are being made to treat this lingering effect [20]. However, chronic pain that begins later in the post-surgical period after weeks or months may be less likely to be recognized and aggressively treated. Notably, FMS tends to be diagnosed only after a long period of persistent pain and failed local treatment.

Although a majority of patients described their pain as worse when they were fatigued, a description often voiced by people with chronic pain, the higher percentage of those with widespread pain who experienced worse pain with after exercise, rest or exposure to cold is similar to those with the specific syndrome of FMS. Increased sensitivity to muscle activity or inactivity as well as sensory input, such as cold, noise, bright lights or smells, are common to people with FMS [29,30]. It appears that women in the widespread pain group resemble those with FMS in some significant ways.

It was interesting and unexpected that those with widespread pain described getting at least some relief from pain medications as many patients with chronic pain get little relief from medication. On the other hand, it is also discouraging to note that many patients with regional pain did not get relief from medication as major strides have been made to aggressively treat neuropathic pain with tricyclic antidepressants and anticonvulsants such as gabapentin [20].

There were few differences in pain descriptions between the two groups with the exception of the emotional impact of pain, which was experienced more, by the widespread pain group. One item, hot-burning, was unexpectedly endorsed more by the widespread pain group, when one might have expected that sensation to be more descriptive of neuropathic pain experienced by the regional pain group.

Both the BPI and the FIQ clearly differentiated the regional from the widespread pain group. As expected, the widespread group was much more impacted. On the FIQ, their overall scores were very close to those noted in FMS studies [42,51]. The regional pain group's scores were much lower and closer to those of rheumatoid arthritis patients who have been used as a comparison group in studies of the psychometric properties of the FIQ [52]. The degree of pain interference with activities and enjoyment of life measured by the BPI was three to four times greater in the widespread pain group when compared to the regional pain group.

Three of the questionnaires, SF-36, FIQ and FACT-B, contain a subscale that focuses on physical functioning or well-being and they are moderately correlated with each other (r = .58–.60). Yet, the FIQ physical activity subscale scores were not significantly different between the two groups while the other two subscales were. The FACT-B physical well-being subscale focuses on symptoms such as fatigue, pain and feeling ill, which would be expected to bother the widespread pain group more. Both the SF-36 and FIQ subscales focus on normal physical activities such as walking, climbing stairs, household activities. However, the SF-36 includes vigorous activities such as walking more than a mile and climbing several flights of stairs, activities that might be much more difficult for a person with widespread pain to accomplish while the FIQ subscale focuses mostly on every day household activities. Thus, it would seem important to clearly identify the contents of subscales such as the ones used in this pilot study before attempting to compare groups of patients on health status and functioning measures.

Although the widespread pain group' total QOLS score was lower than the score of the regional pain group, neither it nor the individual item scores reached statistical significance. The regional pain group's mean total score was as high as those seen in healthy groups while the widespread pain group's score was lower but not as low as the scores seen in untreated FMS patients [34]. The QOLS is an individual satisfaction instrument that focuses on a wide range of domains that are not directly health-related. Scores on the QOLS have been shown to vary more in response to mood and other psychological states than to physical functioning [49]. The widespread pain group had significantly more psychological distress on the measures contained within the FIQ, SF-36 and FACT-B. This may account for the lower QOLS scores that in a larger sample would have reached statistical significance.

There are a number of limitations to this study. First, the sample was one of convenience and cannot be construed as representative of the post-breast cancer surgery population. Second, the sample was small and the number of variables large. Thus, the findings must be viewed with caution as some of the significant differences could have occurred by chance. Third, it is possible that adjuvant therapy rather than the surgery could have caused the chronic pain since both radiation and chemotherapy are known to produce symptoms of fatigue, pain and poor sleep in many patients. However, all patients were at least 1 year post adjuvant therapy and there were no differences in pain severity or other symptoms based on whether or not the subject had gotten adjuvant therapy. In addition, those on current tamoxifen therapy did not differ from those who were not.

This pilot study was carried out primarily to characterize two groups of post-breast cancer surgery patients with different pain patterns. A secondary purpose was to test a set of instruments to see if they would differentiate the two groups. Several of the instruments yielded scores that were significantly different on pain, health status, and interference with multiple aspects of life. This finding points to the need to study chronic pain further after breast cancer surgery and especially attend to the group of women whose pain has become widespread.

## Conclusion

This preliminary work suggests that the women in this study who experienced widespread pain after breast cancer surgery had significantly more pain impact and lower health status than those with regional pain. Further comparative work should be undertaken.

## Authors' contributions

CSB took major responsibility for conceptualization of the project, wrote the proposal, obtained funding, analyzed the data and wrote the complete draft of the paper

KDJ assisted in the literature review and writing of the proposal, oversaw the data collection and data entry, assisted in the analysis of the data, and reviewed drafts of the manuscript. Both authors reviewed the final draft of the manuscript and approved its contents.
